# Genetic Profiling of Glucocorticoid (NR3C1) and Mineralocorticoid (NR3C2) Receptor Polymorphisms before Starting Therapy with Androgen Receptor Inhibitors: A Study of a Patient Who Developed Toxic Myocarditis after Enzalutamide Treatment

**DOI:** 10.3390/biomedicines10061271

**Published:** 2022-05-29

**Authors:** Manuel Morales, Pablo Martín-Vasallo, Julio Ávila

**Affiliations:** 1 Service of Medical Oncology, University Hospital Nuestra Señora de Candelaria, 38010 Santa Cruz de Tenerife, Spain; mmoraleg@ull.edu.es; 2 Laboratorio de Biología del Desarrollo, UD de Bioquímica y Biología Molecular and Centro de Investigaciones Biomédicas de Canarias (CIBICAN), Universidad de La Laguna, 38200 La Laguna, Spain; pmatin@ull.edu.es

**Keywords:** enzalutamide, mineralocorticoid receptor, androgen receptor, androgen receptor inhibitors, glucocorticoid receptor, toxic myocarditis, prostate cancer, aldosterone, spironolactone

## Abstract

Enzalutamide is a nonsteroidal inhibitor of the androgen receptor (AR) signaling pathway and is used to treat patients with metastatic castration-resistant prostate cancer. However, the risk of cardiovascular-related hospitalization in patients with no contraindications for the use of enzalutamide is about 1–2%. To date, the underlying molecular basis of this has not been established. The androgen receptor, glucocorticoid receptor (GR) and mineralocorticoid receptor (MR) are nuclear receptors that share structural similarities and have closely related DNA-binding sites and coregulators. In non-epithelial cells, a fine balance of the activities of these receptors is essential to ensure correct cellular function. In this study, we present a molecular characterization of these nuclear receptors in a prostate cancer patient who developed congestive heart failure after enzalutamide treatment. White cell RNAseq revealed a homozygous rs5522 MR polymorphism and both the rs143711342 and rs56149945 GR polymorphisms, carried in different alleles. No different specific splice isoforms were detected. Recent research suggests that AR inhibition by enzalutamide makes available a coregulator that specifically interacts with the rs5522-mutated MR, increasing its activity and producing adverse effects on cardiovascular health. We suggest an evaluation of the MR rs5522 polymorphism before starting therapy with AR inhibitors.

## 1. Introduction

Enzalutamide is a nonsteroidal antiandrogen that is used to treat patients with metastatic castration-resistant prostate cancer. Enzalutamide has a strong affinity for the androgen receptor (AR) and acts as a competitive inhibitor [1,2,3]. Clinical trials have shown some signs of cardiovascular toxicity, with mainly neurological, cardiac and general disorders such as headache, attention disorders, fatigue, ischemic heart disease or hypertension. In patients with no contraindications or precautions for the use of enzalutamide, the risk of cardiovascular-related hospitalization is about 1–2% [4,5,6]. Following suggestions by several international Urology Society [7] studies on the personalized molecular genetic basis of tumoral pathology and therapy, and considering that the adverse effects of enzalutamide have not been established, here, we report a case of genetic profiling that may be useful for the prevention of the undesirable effects of enzalutamide. For this study, the development of congestive heart failure consecutive to toxic myocarditis [8] in a patient suffering from prostate cancer and undergoing enzalutamide treatment led us to study possible mutations in the mineralocorticoid receptor (MR) based on the improvement in heart function after removing enzalutamide and successful treatment with spironolactone.

The corticosteroid aldosterone controls electrolyte homeostasis in epithelial tissues in organs such as the kidney and intestine. In the heart, it promotes inflammation, frequently leading to fibrosis and/or heart failure [9,10]. Both the physiological and pathophysiological effects of aldosterone are mediated by its binding to the MR, a transcription factor [11]. However, the glucocorticoid cortisol also binds to the MR, with a similar affinity to that of aldosterone. In epithelial tissues, selection for aldosterone is ensured by the cortisol-clearing 11 β-hydroxysteroid dehydrogenase type 2, while in other tissues, such as the heart, cortisol is the primary ligand [12]. This can be explained by the fact that the MR and the glucocorticoid receptor (GR) evolved from a single common ancestor via the duplication of an ancestral corticosteroid receptor gene [13].

The sex steroid hormone receptors androgen receptor (AR), estrogen receptor (ER) and progesterone receptor (PR) are close molecular relatives of the MR and GR and also share pharmacological modulators such as spironolactone and its epoxy derivatives, with variable affinities, tolerability profiles and side effects [14,15]. Enzalutamide is an AR antagonist that not only blocks androgens from binding to their receptors but also inhibits nuclear translocation and binding to DNA [4,16].

The human MR (hMR) consists of 984 amino acids (107 KDa) and has three main domains: the N-terminal domain (NTD), which is responsible for transcriptional activity and is the most structurally divergent between different steroid receptors, the DNA-binding domain (DBD), which consists of two zinc fingers that bind to specific responsive elements (REs) and has the highest degree of homology between different steroid receptors, and finally, the ligand-binding domain (LBD), which is hydrophobic and contains a binding pocket. The LBD is responsible for the high level of promiscuity for different ligands in the steroid receptor family. The MR also has activation function (AF) domains that are capable of binding tissue-specific coregulators. A dimerization domain is found within the DBD [17,18].

The gene coding for the MR is nuclear receptor subfamily 3 group C member 2 (NR3C2) (https://www.ncbi.nlm.nih.gov/gene/4306 (accessed on 22 May 2022)), which is located on chromosome 4 and contains 10 exons within about 450 kb.

In this study, we present evidence that the risk of toxic myocarditis after treatment with androgen receptor inhibitors may be related to MR and GR polymorphisms through increased MR activity elicited by enzalutamide.

## 2. Materials and Methods

### 2.1. Patient’s and Offspring’s Clinical History

This study was approved by the Ethics Committee of the Universidad de La Laguna and Ethical Committee of the Hospital Universitario Nuestra Señora de Candelaria (nº 179; 27 May 2008). The subject and his offspring (2 sons and 1 daughter) signed an informed consent document before entering the project.

Patient’s clinical history: The patient, a 69-year-old Caucasian male, was diagnosed with prostate cancer with multiple bone metastases in August 2015. Complete androgen blockade (LH-RH analog and bicalutamide) was started, achieving a clinical and chemical response. In January 2016, tumor progression was detected, and docetaxel and prednisone were administered for 13 cycles, followed by clinical monitoring and prostate-specific antigen (PSA) testing. In January 2019, after a new PSA elevation, enzalutamide (160 mg/day) was prescribed. After 2 weeks of treatment, the dose of enzalutamide was reduced to 120 mg/day because of grade 3 asthenia. One week later, the patient was hospitalized and diagnosed with congestive heart failure. A transthoracic echocardiogram showed severely depressed left ventricular function with diffuse hypokinesis and a left ventricular ejection fraction (LVEF) of 32%. Magnetic resonance images of the heart were consistent with toxic myocarditis. One week after stopping enzalutamide and being treated with torasemide, spironolactone, ivabradine, ramipril and bisoprolol, the LVEF increased to 53% [8].

At the present time, the patient is undergoing abiraterone/prednisone treatment, is asymptomatic and has PSA levels < 0.01 ng/mL.

To date, offspring have not presented any clinical symptoms that could be related to these mutations.

### 2.2. Leukocyte Isolation

Five-milliliter blood samples were withdrawn and added to an identical volume of PBS 1X (NaH_2_PO_4_ 1.9 mM, Na_2_HPO_4_ 8.1 mM, NaCl 154 mM). The mix was placed over a 5 mL Ficoll-Hypaque cushion (d = 1.077 g/mL) (Sigma-Aldrich, St. Louis, MO, USA) and centrifuged for 30 min at 800× *g*. The intermediate layer formed by mononuclear white cells was gently aspirated, washed three times in three volumes of HBSS (KCl 5.4 mM, Na_2_HPO_4_ 0.3 mM, KH_2_PO_4_ 0.4 mM, NaHCO_3_ 4.2 mM, CaCl_2_ 1.3 mM, MgCl_2_ 0.5 mM, MgSO_4_ 0.6 mM, NaCl 137 mM, D-glucose 5.6 mM, phenol red 0.02%) and centrifuged at 300× *g* for 10 min. The pellet containing cells was resuspended in 1 mL of PBS. Trypan blue testing and cell counting in a Neubauer chamber were performed afterwards. The number of collected cells ranged from 3 to 4 × 10^6^ cells.

### 2.3. White Cell mRNA Extraction and Transcriptome Sequencing

White cells collected from 5 mL of blood were spun at 300× *g* for 5 min. Total RNA from the cellular pellet was extracted using Aurum total RNA mini kit (Bio-Rad Laboratories, Hercules, CA, USA) following the manufacturer’s instructions. Then, 0.5 µg of total RNA was used to construct the RNAseq library and subsequently perform massive RNA sequencing (Servicio de Secuenciación Nucleus, Universidad de Salamanca, Salamanca, Spain). Bioinformatic analysis from the sequencing data was perform by “Servicio de Bioinformática de NUCLEUS” (Universidad de Salamanca). All samples passed the sequencing quality test using FASTQC software [19] (http://www.bioinformatics.babraham.ac.uk/projects/fastqc (accessed on 3 September 2021)) with similar distributions and estimated number of genes expressed.

## 3. Results

### 3.1. Analysis of Genetic Variants of Glucocorticoid and Mineralocorticoid Receptor Genes

**Figure 1:**
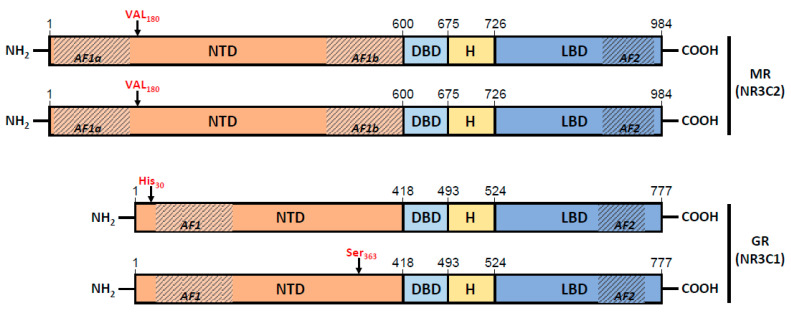
Schematic representation of the protein isoforms expressed by both alleles of MR (NR3C2) and GR (NR3C1) genes in the patient. NTD, N-terminal domain; DBD, DNA-binding domain; H, hinge region; LBD, ligand-binding domain; AF1/AF2, activating function domains 1/2.

Analysis of the patient’s white blood cell mRNA sequences revealed allelic variants of the NR3C1 and NR3C2 genes. The mineralocorticoid receptor (NR3C2) gene was homozygous for genetic variant rs5522, expressing a single protein isoform with Val instead of Ile at position 180 (Table 1). The glucocorticoid receptor (NR3C1) gene showed one different genetic variant in each allele: rs143711342 (Tyr30His) in one allele and rs56149945 (Asn363Ser) in the other allele, confirmed by independent segregation of both mutations in the offspring (Table 1, Figure 1), These results show that the patient’s cells express two different mutated protein isoforms of the glucocorticoid receptor (Table 1).

### 3.2. Analysis of Alternative Splicing of Glucocorticoid and Mineralocorticoid Receptor Genes

**Figure 2:**
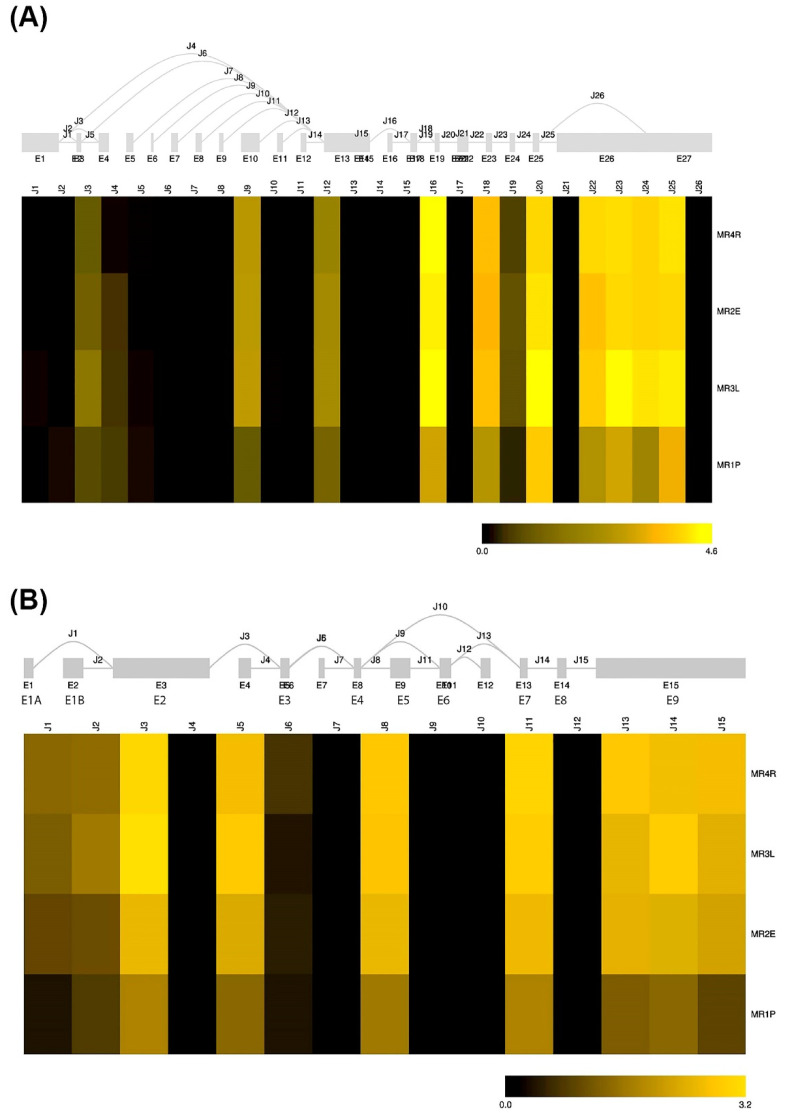
Predicted splice graph and splice junction expression for NR3C1 (**A**) and NR3C2 (**B**). Heatmaps are based on splice junction expression on a log2(FPKM + 1) scale. Grey boxes indicate annotated transcript features. Introns are not drawn to scale.

Analysis of the mRNA sequences with the open-source R/Bioconductor package SGSeq [20] allowed us to predict the existence of different mRNA molecules for the two genes with the alternative use of exon1, as described previously. The percentage of copies of each type of molecule was found to be similar in all samples analyzed (Figure 2). We did not detect specific splice isoforms or shorter molecules that could encode isoforms of truncated proteins.

## 4. Discussion

### 4.1. Mineralocorticoid Receptor: rs5522 Genetic Variant

The genetic variant rs5522 has an isoleucine-to-valine substitution at position 180, which is within the NTD domain and adjacent to the carboxy-terminus of the AF1a subdomain (Figure 1). This region has a considerable degree of disorder in its intrinsic structure, giving it a level of conformational plasticity that allows for the adoption of α-helical structures involved in specific protein–protein interactions or phosphorylation [21]. This structural plasticity permits interactions with various specific proteins that promote induced folding, thus creating new surfaces for further interactions. The N-terminal AF1 and C-terminal AF2 domains are involved in ligand-induced transactivation of the MR [22,23] and in the transactivation of the MR, mediated either by protein–protein interactions at heterologous response elements (HREs) or without direct nuclear receptor binding to DNA at canonical HREs [24].

The AF1 domain is constitutively active and appears to be suppressed by the ligand-free AF2 domain. Binding of the ligand to the C-terminal domain promotes AF2 activation, releasing the suppression of the AF1 domain and thus restoring its function. The AF1 region of the MR is poorly conserved between related nuclear receptors, suggesting that this region may recruit a set of MR-specific coactivators. Moreover, there is evidence that AF1 activity is cell-type-specific and is involved in various tissue-specific actions of synthetic MR ligands [22]. Other studies have shown that the NTD of the MR is involved in the formation of MR–GR heterodimers, which inhibit the transcription of glucocorticoid-sensitive genes, such as those involved in GR-induced apoptosis in the glucocorticoid-sensitive pre-B lymphoma cell line [25].

The change from Ile to Val at position 180 may give rise to subtle differences in the ability to bind to a specific partner that modulates AF1 activity. In Cos7 cells, the recombinant MR isoform with Val180 showed higher activity than the normal isoform when the cells were incubated with 10^−10^ M aldosterone [26]. However, transcriptional activity was significantly lower in the presence of cortisol, suggesting that this polymorphism may affect protein interactions specifically in cortisol-mediated MR effects [27]. Moreover, transfection of HeLa cells with a recombinant MR N-terminal-deletion mutant, including the deletion of amino acids 59–247, led to constitutive MR activity 50% higher than the wild type and a 2.6-fold increase in the level of reporter gene transcription after the addition of aldosterone [28]. These results suggest that this region may contain negative response elements that inhibit the transcription activation function of the N-terminus of the MR [23].

Although the rs5522 mutation is not considered to be a pathological variant, it has been shown to play a role in sporadic pseudohypoaldosteronism [26], hypertension comorbidity in androgen-deprivation therapy [29] and neurological processes such as memory [30] and stress-related disorders [31,32,33]. It is likely that this single polymorphism leads to a slight modulation of MR function, inducing disease only when it co-occurs with certain environmental factors.

### 4.2. Glucocorticoid Receptor: rs143711342 and rs56149945 Genetics Variants

The polymorphisms rs143711342 (Y30H) and rs56149945 (N363S) are located in the NTD domain of the GR, which is responsible for ligand-binding-independent transactivation (AF1 domain). Both are classified in the ClinVar database (NCBI) as variants related to generalized glucocorticoid resistance. The N363S isoform of GR has an altered structure and phosphorylation pattern in the NTD domain that modulates a number of protein regulatory capabilities. In homozygosis, this allelic variant possesses an increased ability to transactivate genes involved in cellular glucocorticoid responses, upregulates IL-15 gene expression and is associated with a higher risk of inflammation in patients with asthma [34] and with higher body mass index and coronary artery diseases [35]. However, there are no previous data about the effect of the Y30H isoform on the GR receptor, and it is currently considered a variant of unknown significance. The presence of the two isoforms in heterozygosis does not lead to alterations of clinical significance. In the present study, each of the two isoforms is expressed by the patient’s alleles. He does not therefore express a wild-type isoform of the GR receptor. The possible synergistic effect of the two mutated isoforms on GR receptor function is currently unknown.

### 4.3. Physiological Effects of GR and MR Genetic Variants in Cardiovascular System

The MR binds cortisol and aldosterone with similar affinity, although the concentration of cortisol in plasma is orders of magnitude higher than that of aldosterone. The aldosterone response in epithelial cells is mediated by the intimate association of the MR with the activity of 11β-hydroxysteroid dehydrogenase type 2 (11βHSD2), which metabolizes cortisol to cortisone, which is unable to bind and activate the MR [36]. However, non-epithelial tissues such as cardiomyocytes, macrophages, immune cells and neural cells do not express this enzymatic activity, so in these cells, the MR actually acts as a second cortisol receptor able to extend the dynamic range for cortisol. The high affinity of the MR for cortisol enables cells to activate a response at low nanomolar concentrations, whereas GR activation requires high nanomolar concentrations [37].

Transgenic mice with GR or/and MR gene inactivation and with MR overexpression have shown different physiological responses. GR knockout mice developed cardiac hypertrophy and left ventricular systolic dysfunction, dying prematurely from heart failure, while MR knockout mice and GR/MR double knockout mice had normal heart morphology and function. Moreover, overexpression of the MR in GR/MR double knockout mice restored the pathophysiological effects previously observed in GR knockout mice [38,39]. Furthermore, in a cardiomyocyte cell line stably expressing the MR, target genes were identified that are involved in extracellular matrix regulation (tenascin-X, ADAMTS1, PAI-1, UPAR and hyaluronic acid synthase-2), signaling and vascular tone regulation (RGS2 and adrenomedullin) and inflammation (orosomucoid) [40].

**Figure 3:**
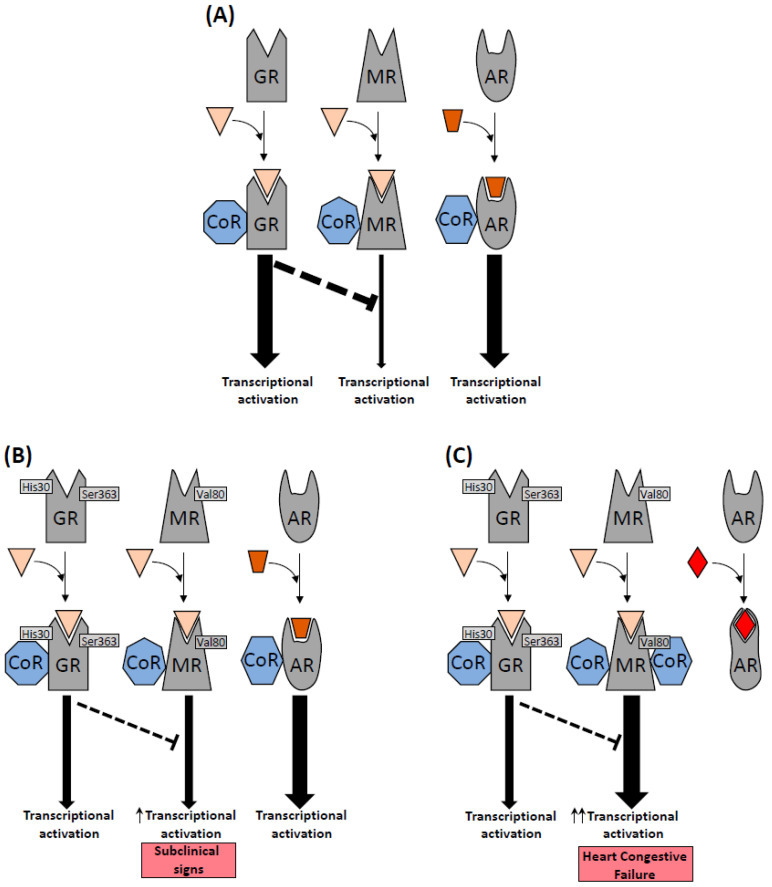
GR, MR and AR signaling pathways in non-epithelial cells. (**A**) Balanced transcriptional activation signaling pathways with normal receptors. (**B**) Increased activation of the MR signaling pathway with expression of MR rs5522 and GR rs143711342/rs56149945. (**C**) Pathological effects of AR inhibition by enzalutamide on the MR signaling pathway in cells expressing MR rs5522 and GR rs143711342/rs56149945.

MR overexpression studies have shown changes in gene expression supporting the hypothesis that balanced cardiomyocyte GR and MR signaling plays a critical role in cardiovascular health (Figure 3), Ca^2+^ handling, oxidative stress, cell death and pro-fibrotic and pro-inflammatory processes [38,39,40,41,42,43,44]. These studies support the idea that the inflammatory component of congestive heart failure is promoted by inappropriate and excessive activation of the MR sustained by glucocorticoid.

MR activity in cells other than cardiomyocytes may also contribute to cardiovascular disease. MR promotes both macrophage and T-lymphocyte activation to the pro-inflammatory M1 and Th1/Th17 phenotypes, thus contributing to cardiovascular fibrosis, which may further contribute to the pathogenesis of cardiac tissue in patients with altered MR activity [45,46].

Taken together, these studies suggest a central role for MR activation in cardiomyocyte hypertrophy and cardiovascular disease, thus supporting the use of the MR antagonists eplerenone and spironolactone in the treatment of patients with heart failure.

### 4.4. MR/GR Polymorphisms and the Effect of Enzalutamide on Cardiac Health

Previous studies on the MR rs5522 polymorphism have shown the stimulation of its function through the effects of the NTD domain [26,28]. This mutation may also affect its association with the GR polymorphisms rs143711342 and rs56149945, increasing the inhibitory effects of the heterodimers on GR action [25].

Patients carrying the rs5522 mutation have been shown to present greater worsening of diastolic function, attenuated by spironolactone treatment [47], as well as left ventricular hypertrophy in patients with resistant hypertension [48], suggesting that the polymorphism contributes to cardiac remodeling.

The patient carrying the rs5522 homozygotic mutation in the present study showed the pathological uptake of a gadolinium tracer in delayed enhancement sequences in the inferior and inferoseptal regions, suggestive of fibrosis, in addition to slightly depressed systolic function. Overall, the findings were compatible with acute myocarditis [8]. Treatment with enzalutamide for 3 weeks caused progressive deterioration in the patient’s functional class, finally leading to acute pulmonary edema. In the kidney, AR receptor inhibition with enzalutamide leads to increased GR and MR activity as a result of increased levels of cellular cortisol caused by specific degradation of 11βHSD2 [49,50]. This unbalanced ratio of AR to GR/MR activity may play an important role in hypokalemia, sodium retention and hypertension observed with enzalutamide treatment.

This adverse effect of enzalutamide, acting as an AR antagonist drug, may also be promoted in cardiovascular tissue by further activation of MR signaling specifically related to the rs5522 polymorphism. Enzalutamide inhibition of AR activity and signaling may lead to the release of coactivators that can specifically bind to the mutated MR, further increasing the activation of the MR signaling pathway and contributing to the subsequent development of congestive heart failure [51].

Recent studies on the therapeutic effects of enzalutamide have shown that the activity of AR signaling was inversely regulated by MR signaling, suggesting a mechanism in which MR competes for DNA-binding sites or coregulators, in addition to transcriptional regulation of the AR [4]. Moreover, there is evidence that the MR and GR share the consensus sequence of the DNA-binding site and coregulators, and that the MR may be a competitive inhibitor of GR that regulates the expression of AR target genes under AR inhibition [4,25]. The association of the rs143711342 and rs56149945 GR polymorphisms (having generalized glucocorticoid resistance) and the rs5522 MR polymorphism (with higher activity than the normal isoform) may produce a synergistic effect that increases MR signaling through the interaction of the mutated MR with a new coregulatory partner released as a result of enzalutamide-induced inhibition of the AR (Figure 3).

In summary, this genetic profiling of a case of toxic myocarditis in enzalutamide therapy is a single input in the transfer of data from basic research and molecular testing to clinical practice, well reviewed in [7]. It represents a particular clinical application of genetic profiling that could be used to select candidates for personalized fine-tuning of oncological therapy in real-life treatment plans, thus potentially reducing costs in terms of money and resources and, above all, avoiding life-threatening circumstances.

## 5. Conclusions

This study reports a clear example of how the genetic profiling of some patients can provide crucial information to allow the fine-tuning of oncological treatment and thus avoid harmful drug treatment side effects. To prevent further cases like this enzalutamide toxic myocarditis, we propose that genetic testing for the MR rs5522 polymorphism be performed before starting therapy with AR inhibitors in prostate cancer, particularly in high-risk patients or patients with comorbidities in organs known to be affected by drugs side effects.

## Figures and Tables

**Table 1:** Genetic variants of glucocorticoid and mineralocorticoid receptors in patient and offspring.

Gene	refSNP	European Allele Frequency	Sample	nt	Allele	aa	Allele
MR (*NR3C2*)	rs5522	A = 0.889731/G = 0.110269	Patient	nt 538	G/G	aa 180	Val/Val
			Son 1		A/G		Ile/Val
			Son 2		A/G		Ile/Val
			Daughter		A/G		Ile/Val
GR (*NR3C1*)	rs143711342	A = 0.999740/G = 0.000260	Patient	nt 88	A/G	aa 30	Tyr/His
			Son 1		A/A		Tyr/Tyr
			Son 2		A/G		Tyr/His
			Daughter		A/A		Tyr/Tyr
GR (*NR3C1*)	rs56149945	T = 0.967909/C = 0.032091	Patient	nt 1088	T/C	aa 363	Asn/Ser
			Son 1		T/C		Asn/Ser
			Son 2		T/T		Asn/Asn
			Daughter		T/C		Asn/Ser

## Data Availability

All data presented in this article can be obtained from the authors upon request.
